# Induced Pluripotent Stem Cells Derived From Two Idiopathic Azoospermia Patients Display Compromised Differentiation Potential for Primordial Germ Cell Fate

**DOI:** 10.3389/fcell.2020.00432

**Published:** 2020-06-25

**Authors:** Fang Fang, Zili Li, Qian Zhao, Zhen Ye, Xiuli Gu, Feng Pan, Honggang Li, Wenpei Xiang, Chengliang Xiong

**Affiliations:** ^1^Institute of Reproductive Health, Tongji Medical College, Huazhong University of Science and Technology, Wuhan, China; ^2^Department of Obstetrics and Gynecology, Union Hospital, Tongji Medical College, Huazhong University of Science and Technology, Wuhan, China; ^3^Wuhan Tongji Reproductive Medicine Hospital, Wuhan, China; ^4^Department of Urology, Union Hospital, Tongji Medical College, Huazhong University of Science and Technology, Wuhan, China

**Keywords:** non-obstructive azoospermia, induced pluripotent stem cells, differentiation, primordial germ cells, transcriptome analysis

## Abstract

At present, the etiology of most non-obstructive azoospermia (NOA) remains unclear. *In vitro* generation of patient-specific induced pluripotent stem cells (iPSCs) is an effective approach for exploring the mechanisms of human disease. Here, we established iPSCs from two patients with idiopathic NOA and differentiated them into primordial germ cell–like cells (PGCLCs) *in vitro*. Compared with iPSCs derived from normal fertile men, the NOA patient-specific iPSCs show decreased efficiency of PGCLC formation *in vitro*. Particularly, the embryoids derived from NOA patient-specific iPSCs show defects in the expression of early primordial germ cell (PGC) genes. The transcriptome analysis reveals the expression patterns of key human PGC genes are generally similar in PGCLCs differentiated from all iPSC lines, and the differentially expressed genes were enriched with gene ontology (GO) of cell cycle and apoptosis regulation. Moreover, the PGCLCs derived from NOA patient-specific iPSCs might have initiated epigenetic reprogramming at a very early stage. Thus, the NOA patient-specific iPSCs exhibit poor response to germ cell induction *in vitro*, which may be related to the regulation of apoptotic process. These findings provide a foundation for future research on mechanism of male infertility.

## Introduction

Infertility is a widespread public health issue, and approximately 10–15% of couples at child-bearing age have difficulties in conceiving (Evers, 2002). Male factor is involved in approximately half of all cases of infertility (Miyamoto et al., 2015). Non-obstructive azoospermia (NOA) is the most severe form of male infertility and characterized by defective spermatogenesis, which is mainly related to genetic factors, such as Y chromosome microdeletions or karyotype abnormalities (An et al., 2018). However, owing to lack of appropriate research models *in vitro* and inaccessibility of early human germline *in vivo*, the genetic etiology remains unclear in most NOA cases, which are diagnosed as idiopathic infertility (Krausz, 2011). Thus, generation of germ cells from human pluripotent stem cells [embryonic stem cells (ESCs) and induced pluripotent stem cells (iPSCs)] could provide a surrogate model for human germline development *in vitro* to better understand the genetic basis of human infertility and also serve as a potential stem cell–based therapy for infertility (Fang et al., 2018a).

It has been demonstrated that primordial germ cell–like cells (PGCLCs) differentiated from mouse ESCs and iPSCs *in vitro* could produce functional gametes and healthy offspring (Hayashi et al., 2011; Hayashi and Saitou, 2013; Hikabe et al., 2016; Zhou et al., 2016). As a result, most of the information about mammalian germ cell development was obtained from mouse model. But evident differences exist between mouse and human germline development, especially for primordial germ cell (PGC) specification (De Felici, 2013; Irie et al., 2014). Recent studies have reported that PGCLCs can be induced *in vitro* from human ESCs (hESCs) and human iPSCs (hiPSCs) in response to signals simulating the natural developing environment of PGC *in vivo* (Kee et al., 2009; Irie et al., 2015; Sasaki et al., 2015). Notably, robust induction of PGCLCs from hiPSCs was established via incipient mesoderm-like cells (iMeLCs) *in vitro* (Sasaki et al., 2015). Based on these differentiation models, several key regulators of human PGC fate as well as the regulation network they formed were identified, including EOMES, SOX17, TFAP2C, and BLIMP1 (Irie et al., 2015; Kojima et al., 2017).

The technology of iPSC has created powerful means to derive patient-specific cells for human disease modeling and also offers promise for personalized cell therapies (Robinton and Daley, 2012). In the field of male infertility, iPSCs have been generated from infertile patients with Klinefelter syndrome or deletions of the Y chromosome azoospermia factor (AZF) regions (Ma et al., 2012; Ramathal et al., 2014; Shimizu et al., 2016). These cells provide a unique platform for mechanism research of male infertility. Notably, Ramathal et al. (2015) introduced the DDX3Y gene into the AZFa-deleted iPSC line and observed a quantifiable improvement in germ cell formation from complemented iPSCs. Furthermore, under a feeder-, serum-, and xeno-free adherent culture condition, Zhao et al. (2018) demonstrated that iPSCs established from patients with NOA showed compromised germ cell development potential.

In this study, we aimed at evaluating the differentiation potential for human germline of idiopathic NOA patient-specific iPSCs. We differentiated the iPSCs of NOA patients into PGCLCs *in vitro* and compared their differentiation potential with normal iPSCs derived from fertile men. Moreover, we performed transcriptome analysis for the differentiating cells during *in vitro* differentiation process to explore the underlying genetic basis of male infertility. Our study creates a research model and also provides insights for future studies on mechanism of male infertility.

## Materials and Methods

The human skin samples were obtained with written informed consent from the infertile and fertile men. The experiments on the induction of human germ cells from hiPSCs/hESCs were approved by the Institutional Review Board of Tongji Medical College, Huazhong University of Science and Technology (S096). All the animal experiments were performed according to the ethical guidelines of Tongji Medical College, Huazhong University of Science and Technology.

### Generation and Culture of iPSCs From Skin Fibroblasts of Idiopathic NOA Patients

The human skin samples were obtained from patients diagnosed as idiopathic NOA and normal fertile men. The dermal fibroblasts were isolated and cultured as described before (Fang et al., 2017). Prior to reprogramming, the dermal fibroblasts were seeded into one well of a six-well culture plate in 2 mL of fibroblast culture medium [Dulbecco modified Eagle medium (DMEM) supplemented with 10% (vol/vol) fetal bovine serum (ESC-qualified) and 0.1 mM non-essential amino acids (NEAA) (all from Life Technologies, Waltham, MA, United States)]. When the cells reached 70–80% confluence, they were infected with OCT4, SOX2, KLF4, and C-MYC retrovirus with 8 μg/mL Polybrene (Sigma, St. Louis, MO, United States). The infection process was repeated 16–24 h later. After 7 days, the cells were passaged with trypsin-EDTA (Thermo Fisher Scientific, Waltham, MA, United States) and plated on inactivated mouse embryonic fibroblast (MEF). The culture medium was changed to hESC medium [knockout DMEM/F12 supplemented with 20% knockout serum replacement (KSR), 2 mM L-glutamine, 0.1 mM NEAA, 55 μM 2-mercaptoethanol and 4 ng/mL recombinant human basic fibroblast growth factor (bFGF) (all from Life Technologies)]. After 21 days, the single colonies with a typical ESC morphology began to appear and were manually picked for expansion on MEF. After approximately 15 passages, the iPSC colonies were transferred to Matrigel (Corning, NY, United States)–coated dishes, and 10 μM ROCK inhibitor (Y-27632, Selleck, Houston, TX, United States) was used for 24 h after plating. All hiPSC lines and hESCs were maintained under a feeder-free condition in mTeSR1 medium (Stem Cell Technologies, Vancouver, BC, Canada).

### Induction of iMeLCs and PGCLCs From Idiopathic NOA Patient-Specific iPSCs

Differentiation of hiPSCs/hESCs into incipient mesoderm-like cells (iMeLCs) and PGCLCs was induced by a protocol published previously (Watanabe et al., 2007; Ying et al., 2008; Sasaki et al., 2015). For iMeLC induction, the hiPSCs/hESCs were dissociated into single cells with a 1 to 1 mixture of TrypLE Select (Life Technologies) and 0.5 mM EDTA/PBS, and 2 × 10^5^ cells were plated into per well of a human plasma fibronectin (Millipore, Burlington, MA, United States)–coated 12-well plate in Glasgow minimum essential medium (GMEM; Life Technologies) supplemented with 15% KSR, 0.1 mM NEAA, 2 mM L-glutamine, 1 mM sodium pyruvate, 0.1 mM 2-mercaptoethanol, 50 ng/mL activin A (ACTA; R&D Systems, Minneapolis, MN, United States), 3 mM CHIR99021 (CHIR, Selleck), and 10 μM ROCK inhibitor (Y-27632). After 2 days of preinduction, approximately 3 × 10^3^ cells were plated into per well of the low-attachment 96-well plate (Corning) in GMEM supplemented with 15% KSR, 0.1 mM NEAA, 2 mM L-glutamine, 1 mM sodium pyruvate, 0.1 mM 2-mercaptoethanol, 200 ng/mL bone morphogenetic protein 4 (BMP4), 100 ng/mL stem cell factor (SCF), 20 ng/mL human leukemia inhibitory factor, 50 ng/mL epidermal growth factor, and 10 μM ROCK inhibitor for PGCLC induction. Media were replaced every day.

### Immunofluorescence Staining

The cells and embryoid bodies (EBs) containing human PGCLCs (hPGCLCs) were fixed in 4% paraformaldehyde for 15 min at room temperature and permeabilized with 1% Triton X-100 (Sigma) for 10 min. After blocking with 10% donkey serum (Jackson ImmunoResearch, West Grove, PA, United States) in phosphate-buffered saline (PBS) for 1 h at room temperature, the samples were incubated with primary antibodies against OCT4 (1:1,000; Abcam, cat. no. ab19857), SSEA4 (1:100; Abcam, cat. no. ab16287), NANOG (1:300; Abcam, Cambridge, MA, United States, cat. no. ab21624), TRA-1-60 (1:500; Abcam, cat. no. ab16288), SOX2 (1:500; Abcam, cat. no. ab97959), TFAP2C (1:400; Cell Signaling Technology, Danvers, MA, United States, cat. no. 2320 or 1:300; Santa Cruz, Dallas, TX, United States, cat. no. sc-12762), BLIMP1 (1:100; R&D Systems, cat. no. MAB36081), and SOX17 (1:500; R&D Systems, cat. no. AF1924) overnight at 4°C, followed by incubation with appropriate AlexaFluor 488– or AlexaFluor 594–conjugated secondary antibodies (1:500; Life Technologies) for 1 h at room temperature the next day. The cell nuclei were counterstained with DAPI (Thermo Fisher Scientific). The samples were evaluated with a Zeiss LSM780 Meta inverted confocal microscope, Carl-Zeiss-Straße 22, Oberkochen, Germany.

### Alkaline Phosphatase Staining

Alkaline phosphatase (AP) activity was tested using AP Staining Kit (Millipore) according to manufacturer’s instructions. The samples were evaluated with a Zeiss LSM780 Meta inverted confocal microscope.

### RNA Isolation and Reverse Transcription–Polymerase Chain Reaction

Total RNA from iPSCs, EBs, and iMeLCs were extracted using Direct-Zol RNA mini-prep (Zymo Research, Irvine, CA, United States) according to manufacturer’s instructions. For EBs formed during PGC induction process, total RNA was extracted using MicroElute Total RNA Kit (Omega, Norcross, GA, United States) according to manufacturer’s instructions. Reverse transcription (RT) was performed using the RevertAid First Strand cDNA synthesis kit (Thermo Fisher Scientific). The RT–polymerase chain reaction (PCR) and quantitative RT-PCR (qRT-PCR) were performed using Premix Taq^TM^ DNA Polymerase and SYBR Premix Ex Taq II (both from Takara, Kusatsu, Shiga, Japan), respectively. Primers are listed in Supplementary Table S1. All gene expression analyses were performed with samples from three independent differentiation experiments.

### Fluorescence-Activated Cell Sorting Analysis

Fluorescence-activated cell sorting (FACS) analysis for pluripotency markers was performed using a Human Pluripotent Stem Cell Transcription Factor Analysis Kit (BD Biosciences, San Jose, CA, United States, cat. no. 560589) according to the manufacturer’s instructions. For PGC surface marker analysis, the hiPSCs were digested using Accutase (Stem Cell Technologies) for 5 min at 37°C, and the iMeLCs and floating aggregates containing hPGCLCs at days 2, 4, 6, and 8 of PGC induction were digested using 0.25% trypsin/EDTA for about 10 min at 37°C. After being washed with PBS supplemented with 0.1% bovine serum albumin (Sigma) for three times, the cell suspensions were filtered through a 40-μm cell strainer (BD Biosciences). The resulting single cells were then incubated with PerCP/Cyanine5.5-conjugated anti-human CD38 (BioLegend, San Diego, CA, United States, cat. no. 356614) and PE/cy7-conjugated anti-human cKIT (BioLegend, cat. no. 313212), or PE-conjugated anti-human EpCAM (eBioscience, Waltham, MA, United States, cat. no. 12-5791-81) and fluorescein isothiocyanate (FITC)–conjugated anti-human INTEGRINα6 (eBioscience, cat. no. 11-0495-82) at 4°C for 30 min. Fluorescence-activated cell sorting analysis was performed using the FACS Calibur system (Becton Dickinson, Franklin Lakes, NJ, United States). The labeled cells were analyzed and sorted by FACSAria^TM^ III (BD Biosciences).

### Embryoid Body Formation

After being incubated with 0.25% trypsin-EDTA, hiPSCs were digested into single cells and cultured in ultralow-attachment dishes (Corning) in hESC culture medium without bFGF. After 3 days of culture, the cells aggregated to form EB. The medium was changed every 2 days. One week later, the EBs in suspension were collected for gene expression analysis.

### Teratoma Formation

Human iPSCs were harvested and injected subcutaneously into the dorsal flanks of 8-week-old male nude mice (1 × 10^6^ cells per mouse). About 8 weeks after injection, teratoma was formed and dissected to be fixed in 4% paraformaldehyde and embedded in paraffin. Sections were stained with hematoxylin–eosin.

### Karyotype Analysis

After culture in medium supplemented with 0.025% colchicine (Sigma) for 8 h, the hiPSCs were subject to hypotonic treatment with 1% sodium citrate for 30 min at room temperature. Then the cells were fixed in freshly prepared methanol/acetic acid (3:1) solution for three times and dropped onto glass slides for chromosome analysis. Chromosomes were visualized by Giemsa staining. Images were captured on a Leica DM 6000B microscope, Buffalo Grove, IL, United States.

### RNA-Sequencing Analysis

For hiPSCs/hESCs and preinduced iMeLCs, total RNAs were extracted with Direct-Zol RNA mini-prep (Zymo Research). The cDNA libraries were prepared using the TruSeq Stranded Total RNA LT Sample Prep Kit (Illumina, San Diego, CA, United States) according to Illumina’s instructions. For EpCAM/INTEGRINα6 double-positive PGCLCs sorted from day 4 embryoids, the full-length cDNA libraries were produced with SMART-Seq v4 Ultra Low Input RNA Kit (Clontech, Mountain View, CA, United States) according to the manufacturer’s instruction. The quality of the library cDNAs was evaluated by Qubit (Invitrogen, Waltham, MA, United States) and Agilent Bioanalyzer 2100 (Agilent, Santa Clara, CA, United States). Sequencing was carried out on Illumina HiSeq2500 according to the manufacturer’s instructions. Published human gonadal PGC data (NCBI GEO: GSE63818) (Guo et al., 2015) were used for unsupervised hierarchical clustering (UHC).

### Bioinformatics Analysis of RNA-Seq

The raw reads were trimmed to remove the adapter sequences and filter the low-quality reads using Trim Galore^1^. The software HTSeq^2^ was used to evaluate the quality of the sequencing data. All the clean reads were mapped to the human genome (GRCh37/hg19) using STAR (Spliced Transcripts Alignment to a Reference) software. The expression levels [fragments per kilobase of transcript per million fragments mapped (FPKM)] were calculated from these mapped reads using the HTSeq and analyzed further in log_2_(FPKM + 1). The UHC and principal component analysis (PCA) were performed in R3.1.1. The software DESeq2 was used to evaluate the differentially expressed genes (DEGs). The DEGs were selected based on the *P* value (<0.05) and the fold changes (>2). To assess the function of the identified DEGs, the Database for Annotation, Visualization and Integrated Discovery (DAVID^3^) v6.7 was used to identify the enriched gene annotations (GO terms).

### Annexin V/Propidium Iodide Assay

The apoptosis rates were measured using a commercial FITC Annexin V Apoptosis Detection Kit (BD Biosciences, cat. no. 556547) according to the manufacturer’s protocol.

### Statistical Analyses

The data were presented as median with quartiles or mean ± standard deviation (SD). Statistical analyses were performed using Wilcoxon signed-ranks test or one-way analysis of variance (ANOVA) with Prism Graphic software. *p* < 0.05 was considered to be statistically significant.

## Results

### Generation and Characterization of hiPSCs Derived From Idiopathic NOA Patients

**FIGURE 1:**
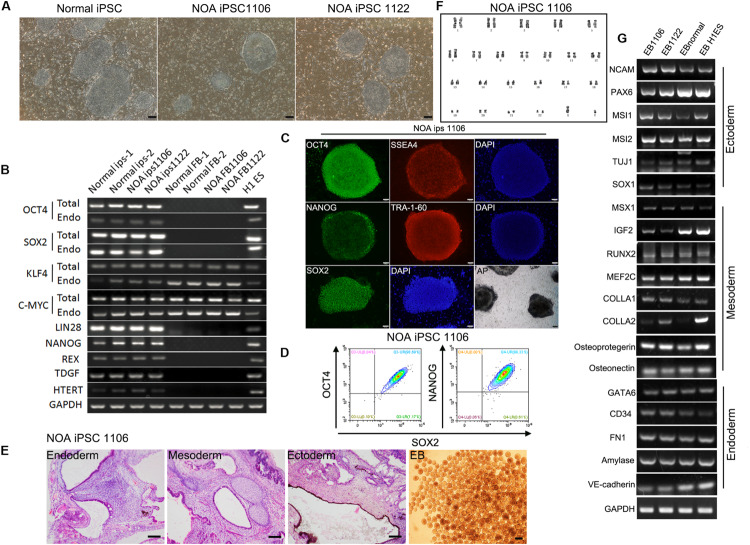
Characterization of hiPSC lines derived from patients with idiopathic non-obstructive azoospermia and normal men. **(A)** Morphology of NOA 1106, NOA 1122, and normal hiPSCs. Scale bar, 200 μm. **(B)** All hiPSC lines express pluripotent genes, H1 ESCs as positive control, and human dermal fibroblasts as negative control. **(C)** NOA 1106 iPSCs show the expression of protein markers for pluripotency. Scale bar, 100 μm. **(D)** Fluorescence-activated cell sorting analysis for OCT4, NANOG, and SOX2 expression in NOA 1106 iPSCs. **(E)** *In vivo* and *in vitro* differentiation of NOA 1106 iPSCs. (Left) Hematoxylin–eosin staining of teratoma sections from NOA 1106 iPSCs shows the evidence of all three germ layers: respiratory epithelium (endoderm), cartilage (mesoderm), and pigmented cells (ectoderm). Scale bar, 100 μm. (Right) Embryoid bodies (EBs) derived from the NOA 1106 iPSCs *in vitro*. Scale bar, 200 μm. **(F)** NOA 1106 iPSCs exhibit normal karyotype in G-band analysis. **(G)** The differentiated cells from the EBs formed by NOA 1106 iPSCs express genes representative of all three germ layers. See also Supplementary Figure S1.

To better understand the mechanism of male infertility, we established hiPSC lines from dermal fibroblasts of two idiopathic NOA patients (NOA 1106 and NOA 1122) and normal fertile men using a method published before (Fang et al., 2018b). The two independent infertile patients were recruited at Wuhan Tongji Reproductive Medicine Hospital following informed consent. They were diagnosed as having idiopathic infertility after routine examination and test for azoospermia. Clinical characteristics are included in Table 1. The hiPSC lines derived in this study have characteristics resembling those of hESCs. They have typical ESC-like morphology, presenting as distinct flat colonies with clear borders (Figure 1A). We confirmed that the hiPSC lines expressed pluripotent genes, and endogenous genes were reactivated (Figure 1B). We also performed immunocytochemistry to examine the expression of ESC markers at protein level. The results showed that these cells were positive for nuclear (OCT4, NANOG, and SOX2) and surface (SSEA4 and TRA1-60) markers of pluripotency, as well as AP (Figure 1C and Supplementary Figure S1A). In addition, flow cytometry analysis for pluripotency markers (OCT4, SOX2, and NANOG) revealed high purity of these obtained hiPSC lines (Figure 1D and Supplementary Figure S1B). Next, we investigated the differentiation potential of the hiPSC lines both *in vitro* and *in vivo*. The ability to form EBs and the expression of genes for the three germ layers were confirmed (Figures 1E,G,I and Supplementary Figure S1D). Moreover, these hiPSC lines were capable of differentiating into the three germ layers (ectoderm, mesoderm, and endoderm) in the teratoma assay (Figure 1E and Supplementary Figure S1D). G banding analysis indicated these hiPSC lines bear normal karyotype (46, XY) (Figure 1F and Supplementary Figure S1C).

**TABLE 1:** Clinical characteristics of the two patients with idiopathic non-obstructive azoospermia.

Characteristics	1106 patient	1122 patient
Age (year)	32	40
FSH (mIU/mL)	8.77	20.1
LH (mIU/mL)	2.53	7.7
Testosterone (ng/mL)	2.45	4.67
Testis size (mL, Rt, Lt)	20, 20	6, 6
Semen analysis	Azoospermia	Azoospermia
AZF deletion	No	No
Cryptorchidism	No	No
Karyotype	Normal	Normal
ART	IVF-D/ET	ICSI/ET
Outcome	Clinical pregnancy	Clinical pregnancy

*FSH, follicle stimulating hormone; LH, luteinizing hormone; Rt, right testis; Lt, left testis; AZF, azoospermia factor; ART, assisted reproductive technique; IVF-D, *in-vitro* fertilization with donor semen; ICSI, intracytoplasmic sperm injection; ET, embryo transfer.*

### Determination of PGCLC Induction Efficiency of NOA Patient-Specific iPSCs

**FIGURE 2:**
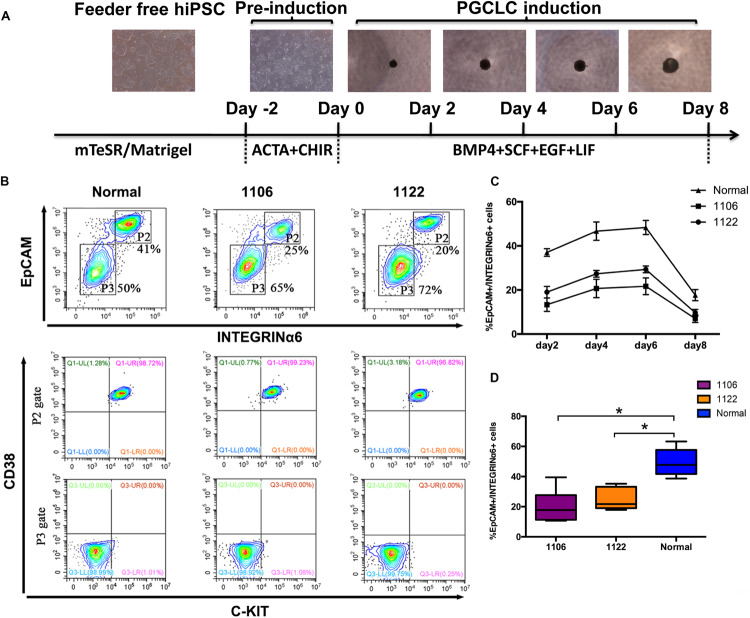
Specification of PGCLCs from idiopathic NOA patient-specific iPSCs. **(A)** Schematic protocol for hPGCLCs specification from idiopathic NOA patient-specific iPSCs and images of hiPSCs, iMeLCs, and floating embryoids containing hPGCLCs. **(B)** (Top)Fluorescence-activated cell sorting analysis by EpCAM and INTEGRINα6 expression of day 4 embryoids differentiated from NOA iPSCs and normal hiPSCs. P2 and P3 gates (boxed areas) indicate EpCAM/INTEGRINa6-high and -low/no cells, respectively. The percentages of cells in the P2 and P3 gates are shown. (Bottom) Fluorescence-activated cell sorting analysis by c-KIT and CD38 of the two populations on the top classified by EpCAM and INTEGRINa6 expression. **(C)** Percentage of EpCAM/INTEGRINα6 double-positive cells in days 2, 4, 6, and 8 floating embryoids determined by FACS. Error bars indicate mean ± SD of three independent experiments. **(D)** Percentage of EpCAM/INTEGRINα6 double-positive cells in day 4 embryoids determined by FACS. The experiments were performed independently for more than six times. Black central line represents the median; boxes represent the 25th and 75th percentiles, and whiskers represent the maximum and minimum. Comparisons were conducted using Wilcoxon signed-ranks test. Asterisk indicates statistically significant differences (*P* < 0.05) between the NOA iPSCs and the normal iPSCs.

Recently, hPGCLCs are robustly induced *in vitro* from hiPSCs in a primed pluripotent state through iMeLCs (Sasaki et al., 2015). With the iMeLC induction method, we here explored the *in vitro* differentiation capacity of idiopathic NOA patient-specific iPSCs into PGCLCs. All hiPSCs, including two patient-specific lines and one normal line, were maintained under a feeder-free condition on Matrigel coating plate. At the same time, one hESC line (H1) was used as control. After 2 days of preinduction, the hiPSCs were differentiated into flat epithelial cells with distinct cell borders (Figure 2A). For PGCLC induction, the differentiating cells were maintained under a floating culture condition and aggregated to form embryoids (Figure 2A).

We performed FACS analysis for the differentiating cells during PGCLC induction process by EpCAM and INTEGRINα6, which were identified as surface markers for hPGCLCs (Sasaki et al., 2015). The proportion of EpCAM/INTEGRINα6 double-positive cells reached the highest at day 4, maintained until day 6, and significantly declined at day 8 (Figure 2C). Interestingly, the average percentage of EpCAM/INTEGRINα6 double-positive cells differentiated from the two independent NOA patient-specific iPSC lines were significantly lower than those derived from the normal hiPSC line (*p* < 0.05) (Figures 2B–D). In general, human gonadal PGCs express the tyrosine kinase receptor c-KIT, which is shared by cells with germline and pluripotent characteristics (Robinson et al., 2001; Høyer et al., 2005; Kerr et al., 2008). Recently, human PGCs in the fetal testes and ovaries have been isolated by FACS using surface marker c-KIT (Gkountela et al., 2013; Guo et al., 2015). Moreover, CD38 could be potentially used as a core marker of human germ cell–related cells (Irie et al., 2015). Hence, we also used the combination of c-KIT and CD38 to sort the EpCAM/INTEGRINα6 double-positive cells. As expected, the EpCAM/INTEGRINα6 double-positive cells were also positive for c-KIT and CD38 (Figure 2B).

### Expression of PGC-Related Genes During hPGCLC Induction From NOA Patient-Specific iPSCs

**FIGURE 3:**
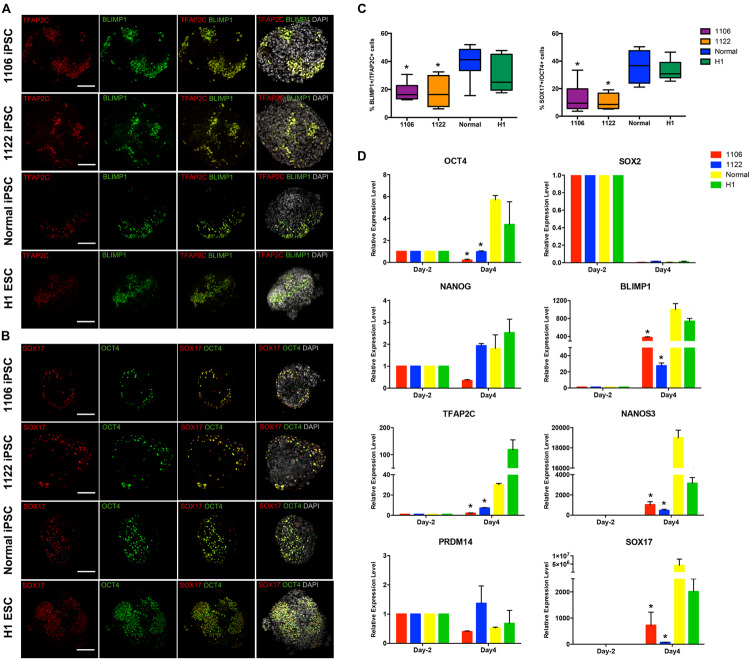
Expression analysis of key genes involved in PGC specification. **(A,B)** Immunofluorescence of day 4 embryoids showing co-expression of TFAP2C with BLIMP1, and OCT4 with SOX17. Scale bars, 100 μm. **(C)** Box plots for the percentage of BLIMP1/TFAP2C (left), and SOX17/OCT4 (right) double-positive cells in day 4 embryoids differentiated from the NOA iPSCs, normal hiPSCs, and hESCs. The quantification of double-positive cells was performed for at least six independent embryoids. Black central line represents the median; boxes represent the 25th and 75th percentiles, and whiskers represent the maximum and minimum, respectively. **(D)** Relative expression levels of PGC-related genes in hiPSCs/hESCs before PGC induction (day -2) and in day 4 embryoids were measured by qRT-PCR and shown with normalization to housekeeping gene GAPDH. Error bars indicate mean ± SD of three independent experiments. Asterisk indicated statistically significant differences (*P* < 0.05) between the NOA iPSCs and the normal iPSCs or H1 ESCs.

Then, we compared the expression of PGC-related genes (BLIMP1, TFAP2C, NANOS3, and SOX17) and pluripotency genes (OCT4, NANOG, and SOX2) at day 4 of the differentiation process between cells differentiated from NOA patient-specific and normal iPSCs. Under the stimulation for PGCLC induction, the day 4 embryoids derived from all four lines initiated significant upregulation of the early PGC genes (BLIMP1, TFAP2C, NANOS3, and SOX17) at mRNA level, but the NOA patient-specific iPSCs exhibited reduced levels compared to normal hiPSCs and hESCs (Figure 3D). The immunofluorescence analysis demonstrated the co-expression of TFAP2C with BLIMP1, as well as OCT4 with SOX17 in day 4 embryoids (Figures 3A,B). Notably, the quantification analysis revealed that the percentage of BLIMP1/TFAP2C and SOX17/OCT4 double-positive cells derived from the NOA patient-specific iPSCs was lower than that from normal cells (Figure 3C). For pluripotency genes, the day 4 embryoids derived from NOA patient-specific iPSCs did not upregulate the expression of OCT4 and NANOG as the normal cells did, whereas the expression of SOX2 was dramatically repressed at day 4 for all the cell lines. At the same time, the expression of PRDM14, a naive pluripotency gene, showed slightly decreased levels after PGCLC induction, except for 1122 NOA iPSCs. These findings indicate that the NOA patient specific-iPSCs respond poorly to PGCLC induction *in vitro*.

### Transcriptome Analysis of PGCLCs Derived From NOA Patient-Specific iPSCs

**FIGURE 4:**
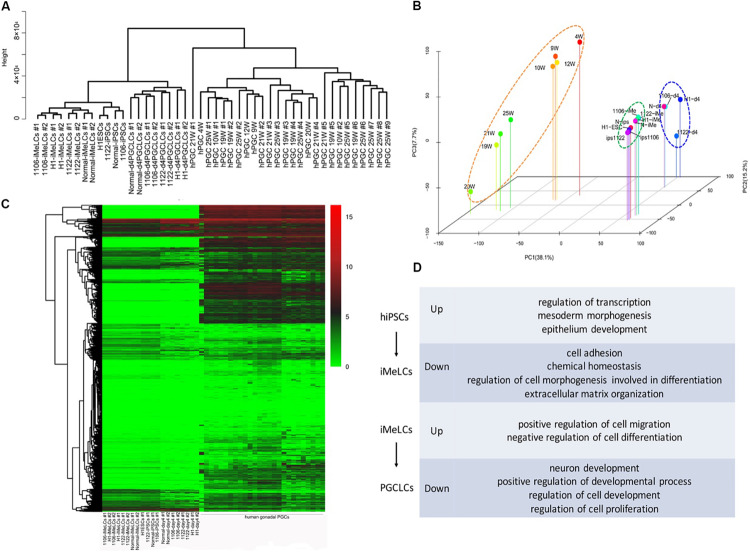
RNA-seq analysis of the PGCLC induction process. **(A)** Unsupervised hierarchical clustering (UHC) of gene expression in hiPSCs, iMeLCs, PGCLCs, and gonadal hPGCs. RNA-seq was performed on two biological replicates (1 and 2) for each cell type. **(B)** PCA of RNA-seq data. **(C)** Heat map of RNA-seq data. **(D)** GO analysis of the genes during hPGCLC specification through iMeLCs.

To examine the global gene expression, we carried out RNA sequencing (RNA-seq) on hiPSCs/hESCs, preinduced iMeLCs, and EpCAM/INTEGRINα6 double-positive PGCLCs sorted from day 4 embryoids. Then we compared the data among different cell lines and also with the transcriptome data of gonadal hPGCs published by Guo et al. (2015). Unsupervised hierarchical clustering of global gene expression classified the cells during *in vitro* differentiation into three clusters, hiPSCs/hESCs, iMeLCs, and PGCLCs at day 4. Notably, iMeLCs formed a subcluster with hiPSCs/hESCs. However, PGCLCs induced from iMeLCs clustered with the branch of hiPSCs/hESCs and iMeLCs first, not gonadal hPGCs in another branch (Figure 4A). Consistently, PCA further described the discrete distribution of cells at different stage. In particular, the gonadal hPGCs settled at the lower extreme of PC1 than the other cell types (Figure 4B). Moreover, the heat map for global gene expression revealed that the majority of the gene expression patterns were different between the induced PGCLCs *in vitro* and the gonadal hPGCs *in vivo* (Figure 4C). We also identified the gene ontology (GO) of biological processes terms that were significantly enriched during PGCLC induction process (Figure 4D).

**FIGURE 5:**
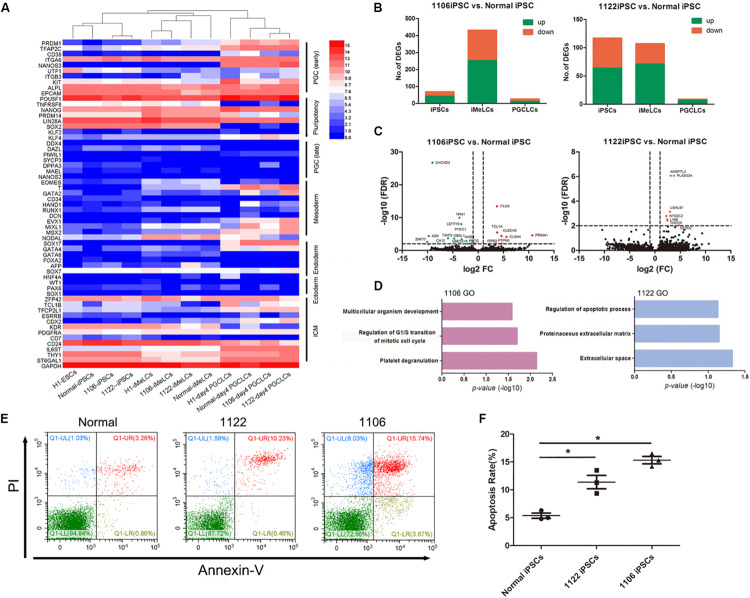
Differentially expressed gene analysis between PGCLCs derived from NOA and normal hiPSCs. **(A)** Heat map of gene expression of key PGC-associated genes (early and late stage) and of pluripotency, mesoderm, endoderm, ectoderm, and ICM genes. **(B)** Numbers of genes upregulated or downregulated at key stages during PGCLC specification of NOA 1106 iPSCs (left) and NOA 1122 iPSCs (right) compared with that of normal iPSCs. **(C)** Volcano plot of the DEGs in PGCLCs derived from NOA 1106 iPSCs (left) and NOA 1122 iPSCs (right) compared with PGCLCs derived from normal iPSCs. The red dots indicate genes upregulated, and the green dots indicate genes downregulated. **(D)** Enriched GO terms in the upregulated genes of PGCLCs derived from NOA 1106 iPSCs (left) and NOA 1122 iPSCs (right) compared with PGCLCs derived from normal iPSCs. Primordial germ cell–like cells, the EpCAM, and INTEGRINα6 double-positive cells in day 4 embryoids. **(E)** Fluorescence-activated cell sorting analysis by annexin V and PI staining for day 4 embryoids differentiated from NOA iPSCs and normal hiPSCs. The percentages of cells in the four quadrants are shown. **(F)** Apoptotic rates of day 4 embryoids differentiated from NOA iPSCs and normal hiPSCs. Error bars indicate mean ± SD of three independent experiments. Comparisons were conducted using ANOVA. Asterisk indicated statistically significant differences (*P* < 0.05) between the NOA iPSCs and the normal iPSCs.

Next, we selected the representative genes associated with pluripotency, PGC specification, inner cell mass (ICM), and embryonic lineage development and analyzed their expression profiles among these cell types in detail. The expression patterns of these genes during PGCLC induction process from all cell lines were generally similar (Figure 5A). Genes for early PGC (PRDM1, TFAP2C, KIT, and NANOS3) were upregulated in PGCLCs, whereas genes for late PGC (DDX4, DAZL, PIWIL1, and SYCP3) still exhibited low expression. Approximately half of genes related to pluripotency and ICM were still highly expressed in PGCLCs, such as OCT4, NANOG, TFCP2L1, and KLF4; however, compared with hiPSCs and iMeLCs, the expression of PRDM14, SOX2, and TNFRSF8 was downregulated in PGCLCs. Moreover, the expression of mesoderm gene T and endoderm gene SOX17 was upregulated in PGCLCs. Notably, some of the mesoderm genes (EOMES, RUNX1, and NODAL) were upregulated in iMeLCs, but further downregulated in PGCLCs.

Then, we evaluated the DEGs between PGCLCs induced from NOA patient-specific iPSCs and normal hiPSCs. Compared with normal line, the numbers of upregulated or downregulated genes for 1106 line at iMeLC stage were substantially increased, whereas the numbers for 1122 line were slightly decreased at the same stage. As for the PGCLC stage, the numbers of DEGs for both 1106 and 1122 lines were reduced to minimum (Figure 5B). Specifically, we identified the DEGs between the PGCLCs derived from NOA patient-specific iPSCs and normal hiPSCs (Figure 5C). The DEGs for 1106 line were enriched with GO terms such as multicellular organism development, regulation of G1/S transition of mitotic cell cycle, and platelet degranulation. And the DEGs for 1122 line were enriched with GO terms such as regulation of apoptotic process, proteinaceous extracellular matrix, and extracellular space (Figure 5D). In consideration of the DEGs involved in cell cycle and regulation of apoptosis, we performed annexin V/propidium iodide (PI) staining for the day 4 embryoids to evaluate the apoptosis rates. Consistently, the percentage of apoptotic cells differentiated from NOA patient-specific iPSCs were higher than those from normal hiPSCs (Figures 5E,F).

Additionally, we analyzed the expression of key epigenetic regulators involved in DNA methylation and demethylation, DNA methyltransferases (DNMTs), UHRF1, and TETs. With the sequencing data, we observed that the hPGCLCs derived from all hiPSC lines presented similar expression patterns for epigenetic regulators. For TET family members involved in DNA demethylation, hPGCLCs showed slightly increased expression of TET1 and TET2 (Supplementary Figure S2). Meanwhile, hPGCLCs repressed the expression of DNMT3A, DNMT3B, and UHRF1, but retained the expression of DNMT1 (Supplementary Figure S2).

Collectively, the transcriptome of PGCLCs derived from NOA patient-specific iPSCs *in vitro* is distinct from that of *in vivo* gonadal hPGCs, and DEG analysis indicated that the regulation of apoptosis might be involved in the limited differentiation potential of NOA patient-specific iPSCs. Moreover, the hPGCLCs differentiated from idiopathic NOA patient-specific iPSCs *in vitro* might have initiated global epigenetic reprogramming, albeit at a very early stage.

## Discussion

In this study, we differentiated the idiopathic NOA patient-specific iPSCs into PGCLCs *in vitro* with a two-step method simulating the natural developing environment *in vivo*. Compared with the normal hiPSCs, the idiopathic NOA patient-specific iPSCs showed decreased efficiency for PGC induction *in vitro* and defects in the expression of key genes involved in hPGC specification. Further transcriptome analysis for *in vitro* differentiation process revealed that the expression patterns of key genes involved in human PGC specification are generally similar in the PGCLCs derived from all iPSC lines, but most of the transcriptomes of PGCLCs derived *in vitro* are quite distinct from those of embryonic gonadal PGCs *in vivo*. Moreover, the biological functions of DEGs between PGCLCs derived from azoospermic patients and fertile men were related to the regulation of cell cycles and apoptosis. All these defects might be related to the genetic causes for male infertility.

In mammals, the induction of gene regulatory network in germline-competent cells for PGC specification requires signals from extraembryonic tissues, mainly involving BMP and WNT signals (Ohinata et al., 2009; Nikolic et al., 2016; Tang et al., 2016). In response to BMP and WNT signaling, mouse PGC specification is initiated with the expression of BLIMP1 (also known as PRDM1), which is followed by the upregulation of PRDM14 and TFAP2C (Yamaji et al., 2008). However, PRDM14 plays a less prominent role in human germline development (Guo et al., 2015; Sugawa et al., 2015). In accordance with this, we observed that the expression of PRDM14 was not activated during the *in vitro* PGC specification from hiPSCs both at embryoid level (qPCR) and single-cell level (RNA-seq). Moreover, the pluripotency network is re-established during PGC development, and mPGCs express the core pluripotency gene SOX2 (Grabole et al., 2013), which became negative in the derived hPGCLCs *in vitro*. Notably, SOX17 is a critical regulator of hPGC fate and is upstream of BLIMP1 expression, which represses the endodermal and other somatic genes during hPGCLC specification from hESCs (Irie et al., 2015). WNT signals induce the expression of EOMES to activate SOX17, which works together with TFAP2C to instate the hPGCLC transcriptional program, including the BLIMP1 expression (Kojima et al., 2017). Particularly, the day 4 embryoids differentiated from NOA patient-specific iPSCs exhibited decreased level of BLIMP1, TFAP2C, and SOX17. Thus, the repressed gene regulatory network of hPGC specification may play an important role in the early mechanism of idiopathic NOA.

Recently, epigenetic modifications have been suggested to be involved in the etiology of male idiopathic infertility, such as DNA methylation, modification of histones, and non-coding RNAs (Carrell, 2012; Laurentino et al., 2016). In mammals, global epigenetic reprogramming occurs in the early germline to erase parental epigenetic memories and facilitate gametogenesis. The regulatory network of PGC fate drives comprehensive DNA demethylation by repressing DNA methylation and activating TET-mediated hydroxymethylation (Tang et al., 2015). Furthermore, hPGCs exhibit transiently high levels of 5hmC, which are coupled with the upregulation of TET1 and TET2 (Tang et al., 2015). In humans, the gene expression program of PGC (SOX17, BLIMP1, and TFAP2C) only inhibits the expression of DNMT3B, whereas in mice the expression of DNMT3A and DNMT3B is both repressed after PGC specification (Kojima et al., 2017). However, the RNA-seq data reveal that hPGCLCs derived from hiPSCs *in vitro* also downregulated DNMT3A slightly in addition to significant repression of DNMT3B. In particular, Sosa et al. (2018) reported that rhesus macaque PGCLCs (rPGCLCs) generated *in vitro* correspond to SOX17/TFAP2C/PRDM1 newly specified rPGCs *in vivo* that have not initiated global 5mC/5hmC epigenetic reprogramming. Thus, we speculated that the hPGCLCs differentiated from idiopathic NOA patient-specific iPSCs *in vitro* might have initiated epigenetic reprogramming at a very early stage and not as completely as the late PGCs specified *in vivo*.

However, the main limitation was that we established iPSC lines from two NOA patients, and it would be meaningful to generate more patient-specific iPSC lines for research on mechanism of male infertility. Moreover, it is also possible that the key defects in spermatogenesis in these NOA patients occur at a late stage after PGC formation, which still presents difficulties for modeling with hiPSCs *in vitro*.

## Conclusion

Compared with normal hiPSCs, the NOA patient-specific iPSCs exhibit poor response to PGCLC induction *in vitro*, and the compromised differentiation potential for germ cell fate might be correlated with apoptosis mechanism. Moreover, PGCLC specification *in vitro* cannot completely reconstitute the development of gonadal PGCs *in vivo*, which also depends on signals from surrounding somatic cells. Therefore, more comprehensive exploration on human germ cell development remains to be implemented for better understanding of idiopathic infertility.

## Data Availability Statement

The datasets generated for this study can be found in the NCBI Gene Expression Omnibus (NCBI GEO: GSE126009).

## Ethics Statement

The studies involving human participants were reviewed and approved by the Institutional Review Board of Tongji Medical College, Huazhong University of Science and Technology. The patients/participants provided their written informed consent to participate in this study. The animal study was reviewed and approved by the Institutional Review Board of Tongji Medical College, Huazhong University of Science and Technology. Written informed consent was obtained from the individual(s) for the publication of any potentially identifiable images or data included in this article.

## Author Contributions

The study was conceived and designed by FF, ZL, and CX. FF and ZL conducted the majority of experiments and analyzed the data. QZ contributed to the generation of NOA iPSC lines and immunofluorescence staining. ZY and FP conducted the patient recruitment and consents, and clinical sample collection. XG performed the karyotype analysis. FF wrote the manuscript. The study was supervised by CX, HL, and WX. The final version was approved by all authors.

## Conflict of Interest

The authors declare that the research was conducted in the absence of any commercial or financial relationships that could be construed as a potential conflict of interest.

## Footnotes

1 www.bioinformatics.babraham.ac.uk/projects/trim_galore

2 https://pypi.python.org/pypi/HTSeq

3 https://david.ncifcrf.gov

## Supplementary Material

FIGURE S1 Characterization of hiPSC lines derived from patients with idiopathic non-obstructive azoospermia and normal men, related to Figure 1. **(A)** Both NOA 1122 and normal iPSCs show the expression of protein markers for pluripotency. Scale bar, 100 μm. **(B)** FACS analysis for OCT4, NANOG and SOX2 expression in NOA 1122 and normal iPSCs. **(C)** Both NOA 1122 and normal iPSCs exhibit normal karyotype in G-band analysis. **(D)** *In vivo* and *in vitro* differentiation of NOA 1122 and normal iPSCs. (Left) Hematoxylin and eosin staining of teratoma sections shows the evidence of all three germ layers: respiratory epithelium (endoderm), cartilage (mesoderm), and pigmented cells (ectoderm). Scale bar, 100 μm. (Right) Embryoid bodies (EBs) formation *in vitro*. Scale bar, 200 μm.

FIGURE S2 Expression analysis of methylation related epigenetic modifiers from RNA-seq data. Mean normalized read counts from two biological replicates were shown during PGC induction from NOA 1106 iPSCs **(A)**, NOA 1122 iPSCs **(B)**, and normal iPSCs **(C)** through iMeLCs.
