# Corrigendum: Induced Pluripotent Stem Cells Derived From Two Idiopathic Azoospermia Patients Display Compromised Differentiation Potential for Primordial Germ Cell Fate

**DOI:** 10.3389/fcell.2020.00718

**Published:** 2020-08-25

**Authors:** Fang Fang, Zili Li, Qian Zhao, Zhen Ye, Xiuli Gu, Feng Pan, Honggang Li, Wenpei Xiang, Chengliang Xiong

**Affiliations:** ^1^Institute of Reproductive Health, Tongji Medical College, Huazhong University of Science and Technology, Wuhan, China; ^2^Department of Obstetrics and Gynecology, Union Hospital, Tongji Medical College, Huazhong University of Science and Technology, Wuhan, China; ^3^Wuhan Tongji Reproductive Medicine Hospital, Wuhan, China; ^4^Department of Urology, Union Hospital, Tongji Medical College, Huazhong University of Science and Technology, Wuhan, China

**Keywords:** non-obstructive azoospermia, induced pluripotent stem cells, differentiation, primordial germ cells, transcriptome analysis

In the published article, there was an error in affiliations 1 and 2. Affiliation 1 should be “Institute of Reproductive Health, Tongji Medical College, Huazhong University of Science and Technology, Wuhan, China,” and affiliation 2 should be “Department of Obstetrics and Gynecology, Union Hospital, Tongji Medical College, Huazhong University of Science and Technology, Wuhan, China.”

The authors apologize for this error and state that this does not change the scientific conclusions of the article in any way. The original article has been updated.

