# Supplementary figures and images for: Induced Pluripotent Stem Cells Derived From Two Idiopathic Azoospermia Patients Display Compromised Differentiation Potential for Primordial Germ Cell Fate
Source: Front Cell Dev Biol. 2020 Jun 25;8:432. doi: 10.3389/fcell.2020.00432 (PMC7331483; doi:10.3389/fcell.2020.00432)

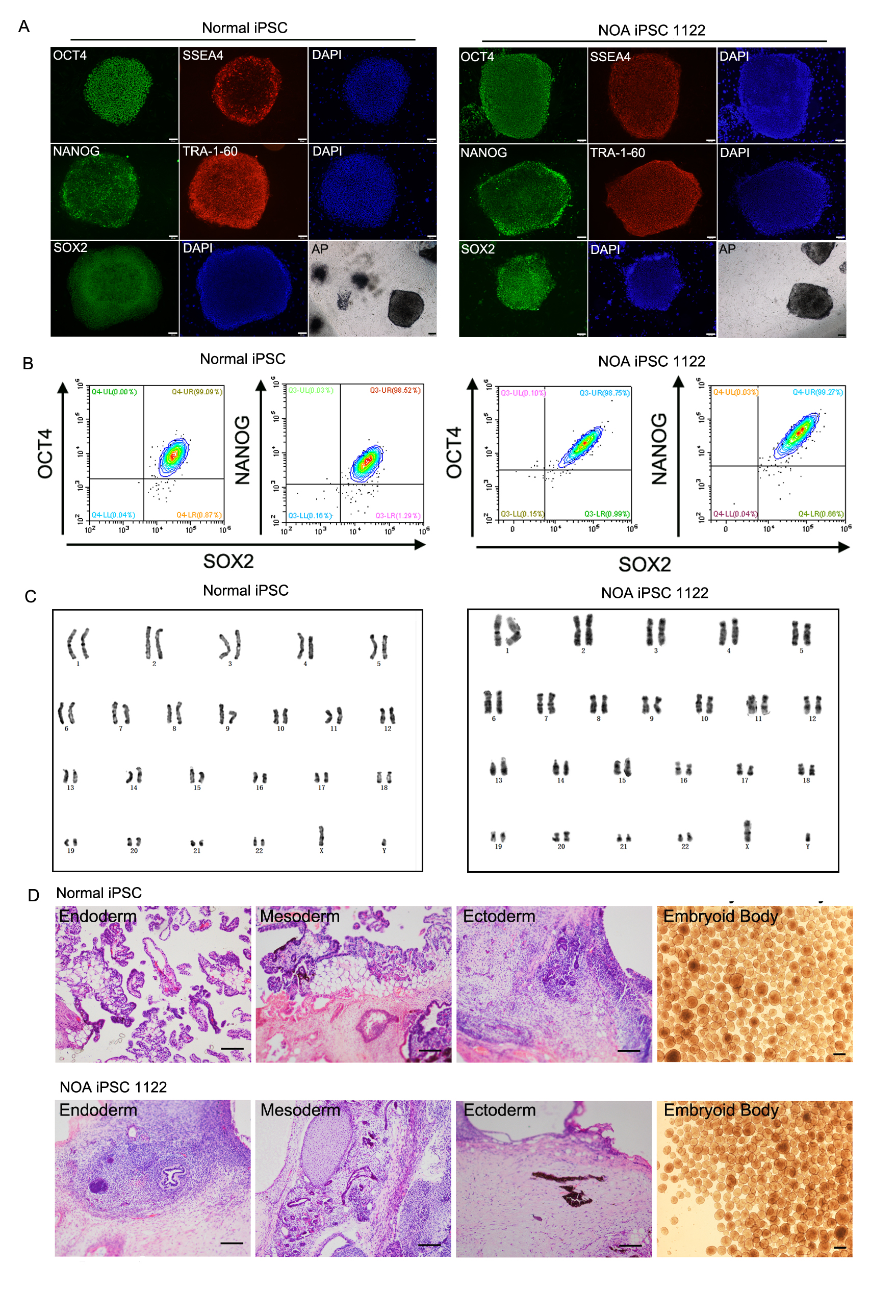

Supplement: FIGURE S1 — Characterization of hiPSC lines derived from patients with idiopathic non-obstructive azoospermia and normal men, related to Figure 1. (A) Both NOA 1122 and normal iPSCs show the expression of protein markers for pluripotency. Scale bar, 100 μm. (B) FACS analysis for OCT4, NANOG and SOX2 expression in NOA 1122 and normal iPSCs. (C) Both NOA 1122 and normal iPSCs exhibit normal karyotype in G-band analysis. (D) In vivo and in vitro differentiation of NOA 1122 and normal iPSCs. (Left) Hematoxylin and eosin staining of teratoma sections shows the evidence of all three germ layers: respiratory epithelium (endoderm), cartilage (mesoderm), and pigmented cells (ectoderm). Scale bar, 100 μm. (Right) Embryoid bodies (EBs) formation in vitro. Scale bar, 200 μm. [file Image_1.TIF]

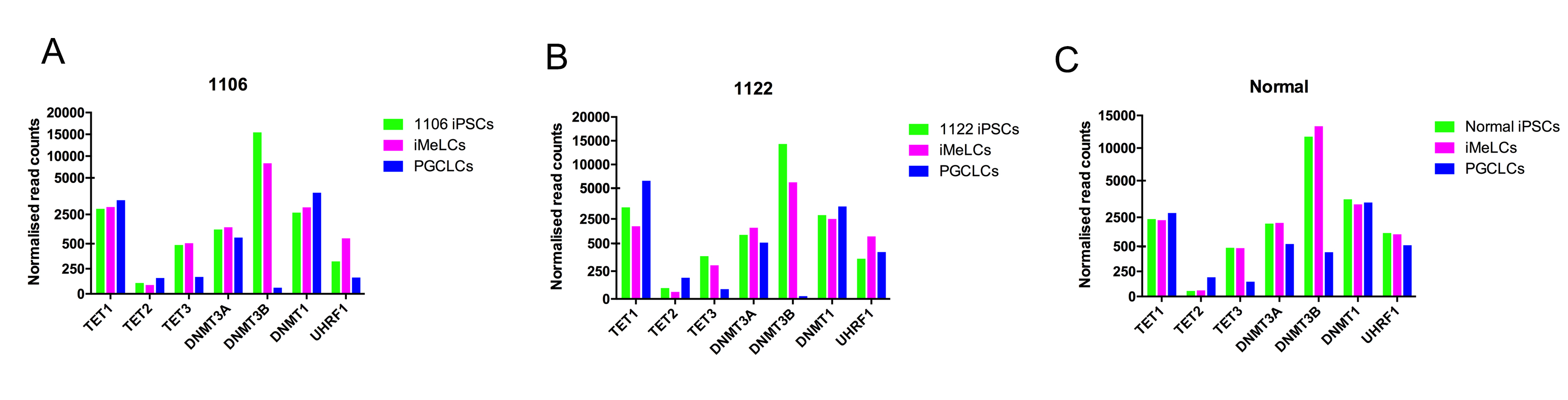

Supplement: FIGURE S2 — Expression analysis of methylation related epigenetic modifiers from RNA-seq data. Mean normalized read counts from two biological replicates were shown during PGC induction from NOA 1106 iPSCs (A), NOA 1122 iPSCs (B), and normal iPSCs (C) through iMeLCs. [file Image_2.TIF]
