# Supplementary material for: Induced Pluripotent Stem Cells Derived From Two Idiopathic Azoospermia Patients Display Compromised Differentiation Potential for Primordial Germ Cell Fate
Source: Front Cell Dev Biol. 2020 Jun 25;8:432. doi: 10.3389/fcell.2020.00432 (PMC7331483; doi:10.3389/fcell.2020.00432)
Supplement: Supplementary file 3 [file Table_1.DOCX]

**Table S1**. Primers used in this study

| Gene | Primer sequence (5'-3') |
| --- | --- |
| Total-OCT4 Forward | GTGTTCAGCCAAAAGACCATCT |
| Total-OCT4 Reverse | GGCCTGCATGAGGGTTTCT |
| Total-SOX2 Forward | GCCGAGTGGAAACTTTTGTCG |
| Total-SOX2 Reverse | GGCAGCGTGTACTTATCCTTCT |
| Total-C-MYC Forward | GTCAAGAGGCGAACACACAAC |
| Total-C-MYC Reverse | TTGGACGGACAGGATGTATGC |
| Total-KLF4 Forward | CGGACATCAACGACGTGAG |
| Total-KLF4 Reverse | GACGCCTTCAGCACGAACT |
| NANOG Forward | ACAACTGGCCGAAGAATAGCA |
| NANOG Reverse | GGTTCCCAGTCGGGTTCAC |
| LIN28 Forward | GTTCGGCTTCCTGTCCAT |
| LIN28 Reverse | CTGCCTCACCCTCCTTCA |
| OCT4 (Endogenous) Forward | GACAGGGGGAGGGGAGGAGCTAGG |
| OCT4 (Endogenous) Reverse | CTTCCCTCCAACCAGTTGCCCCAAAC |
| SOX2 (Endogenous) Forward | GGGAAATGGGAGGGGTGCAAAAGAGG |
| SOX2 (Endogenous) Reverse | TTGCGTGAGTGTGGATGGGATTGGTG |
| KLF4 (Endogenous) Forward | ACGATCGTGGCCCCGGAAAAGGACC |
| KLF4 (Endogenous) Reverse | TGATTGTAGTGCTTTCTGGCTGGGCTCC |
| C-MYC (Endogenous) Forward | GCGTCCTGGGAAGGGAGATCCGGAGC |
| C-MYC (Endogenous) Reverse | TTGAGGGGCATCGTCGCGGGAGGCTG |
| HTERT Forward | TACTTTGTCAAGGACAGGCTCA |
| HTERT Reverse | CAGGTGAGCCACGAACTGTC |
| REX1 Forward | CTAGGCAAACCCACCCCACT |
| REX1 Reverse | TTCAGCAAACACCTGCTGGAC |
| TDGF Forward | TGCTACGACCTTCTGGGGAA |
| TDGF Reverse | AATCACACTGTAAGAGAAGCGG |
| NCAM Forward | AGGAGACAGAAACGAAGCCA |
| NCAM Reverse | GGTGTTGGAAATGCTCTGGT |
| PAX6 Forward | GCCAGCAACACACCTAGTCA |
| PAX6 Reverse | TGTGAGGGCTGTGTCTGTTC |
| SOX1 Forward | GGGAAAACGGGCAAAATAAT |
| SOX1 Reverse | CCATCTGGGCTTCAAGTGTT |
| MSI1 Forward | ACCCCCACATTCTCTCACTG |
| MSI1 Reverse | AAACCCAAAACACGAACAGC |
| MSI2 Forward | TTTGTAGGCGGGTTATCTGC |
| MSI2 Reverse | GCCATAGCTTGGAGCAAATC |
| TUJ1 Forward | ACCTCAACCACCTGGTATCG |
| TUJ1 Reverse | GGGTACCACTCCACGAAGTA |
| MSX1 Forward | TCCTCAAGCTGCCAGAAGAT |
| MSX1 Reverse | TACTGCTTCTGGCGGAACTT |
| IGF2 Forward | CAGACCCCCAAATTATCGTG |
| IGF2 Reverse | GCCAAGAAGGTGAGAAGCAC |
| COLLA1 Forward | GGACACAATGGATTGCAAGG |
| COLLA1 Reverse | TAACCACTGCTCCACTCTGG |
| COLLA2Forward | CCGCGGRGAGCCATGATTCG |
| COLLA2Reverse | CAGGCCCAGGAGGTCCTTTGGG |
| RUNX2 Forward | CGGCAAAATGAGCGACGTG |
| RUNX2 Reverse | CACCGAGCACAGGAAGTTG |
| Osteoprotegerin Forward | AGCACCCTGTAGAAAACACAC |
| Osteoprotegerin Reverse | ACACTAAGCCAGTTAGGCGTAA |
| Osteonectin Forward | AGCACCCCATTGACGGGTA |
| Osteonectin Reverse | GGTCACAGGTCTCGAAAAAGC |
| MEF2CForward | AGATACCCACAACACACCACGCGCC |
| MEF2CReverse | ATCCTTCAGAGAGTCGCATGC |
| GATA6 Forward | CCATGACTCCAACTTCCACC |
| GATA6 Reverse | ACGGAGGACGTGACTTCGGC |
| Amylase Forward | GCTGGGCTCAGTATTCCCCAAAT |
| Amylase Reverse | GACGACAATCTCTGACCTGAGTAG |
| FN1 Forward | CCACTTCCCCTTCCTATACAAC |
| FN1 Reverse | ACGACCATTCCCAACACAC |
| CD34 Forward | AAATCCTCTTCCTCTGAGGCTGGA |
| CD34 Reverse | AAGAGGCAGCTGGTGATAAGGGTT |
| VE-cadherin Forward | TGGAGAAGTGGCATCAGTCAACAG |
| VE-cadherin Reverse | TCTACAATCCCTTGCAGTGTGAG |
| BLIMP1 Forward | AAACCAAAGCATCACGTTGACA |
| BLIMP1Reverse | GGATGGATGGTGAGAGAAGCAA |
| TFAP2C Forward | ATTAAGAGGATGCTGGGCTCTG |
| TFAP2CReverse | CACTGTACTGCACACTCACCTT |
| NANOS3 Forward | TGGCAAGGGAAGAGCTGAAATC |
| NANOS3Reverse | TTATTGAGGGCTGACTGGATGC |
| PRDM14Forward | TATCATACTGTGCACTTGGCAGAA |
| PRDM14Reverse | AGCAACTGGGACTACAGGTTTGT |
| SOX17 Forward | TTCGTGTGCAAGCCTGAGAT |
| SOX17Reverse | TAATATACCGCGGAGCTGGC |
| GAPDH Forward | TGAAGGGTGGAGCCAAAAG |
| GAPDH Reverse | AGTCTTCTGGGTGGCAGTGAT |
